# Assessment of the accuracy of imaging software for 3D rendering of the upper airway, usable in orthodontic and craniofacial clinical settings

**DOI:** 10.1186/s40510-022-00413-8

**Published:** 2022-06-13

**Authors:** Antonino Lo Giudice, Vincenzo Ronsivalle, Giorgio Gastaldi, Rosalia Leonardi

**Affiliations:** 1 Department of General Surgery and Medical-Surgical Specialties, School of Dentistry, Unit of Orthodontics, University of Catania, Policlinico Universitario “Gaspare Rodolico - San Marco”, Via Santa Sofia 78, 95123 Catania, Italy; 2 Department Orthodontics, Vita-Salute San Raffaele University, Milan, Italy

**Keywords:** 3D rendering, Upper airway, OSAS, Cone-beam computed tomography, Orthodontics

## Abstract

**Background:**

Several semi-automatic software are available for the three-dimensional reconstruction of the airway from DICOM files. The aim of this study was to evaluate the accuracy of the segmentation of the upper airway testing four free source and one commercially available semi-automatic software. A total of 20 cone-beam computed tomography (CBCT) were selected to perform semi-automatic segmentation of the upper airway. The software tested were Invesalius, ITK-Snap, Dolphin 3D, 3D Slicer and Seg3D. The same upper airway models were manually segmented (Mimics software) and set as the gold standard (GS) reference of the investigation. A specific 3D imaging technology was used to perform the superimposition between the upper airway model obtained with semi-automatic software and the GS model, and to perform the surface-to-surface matching analysis. The accuracy of semi-automatic segmentation was evaluated calculating the volumetric mean differences (mean bias and limits of agreement) and the percentage of matching of the upper airway models compared to the manual segmentation (GS). Qualitative assessments were performed using color-coded maps. All data were statistically analyzed for software comparisons.

**Results:**

Statistically significant differences were found in the volumetric dimensions of the upper airway models and in the matching percentage among the tested software (*p* < 0.001). Invesalius was the most accurate software for 3D rendering of the upper airway (mean bias = 1.54 cm^3^; matching = 90.05%) followed by ITK-Snap (mean bias =  − 2.52 cm^3^; matching = 84.44%), Seg 3D (mean bias = 3.21 cm^3^, matching = 87.36%), 3D Slicer (mean bias =  − 4.77 cm^3^; matching = 82.08%) and Dolphin 3D (difference mean =  − 6.06 cm^3^; matching = 78.26%). According to the color-coded map, the dis-matched area was mainly located at the most anterior nasal region of the airway. Volumetric data showed excellent inter-software reliability (GS vs semi-automatic software), with coefficient values ranging from 0.904 to 0.993, confirming proportional equivalence with manual segmentation.

**Conclusion:**

Despite the excellent inter-software reliability, different semi-automatic segmentation algorithms could generate different patterns of inaccuracy error (underestimation/overestimation) of the upper airway models. Thus, is unreasonable to expect volumetric agreement among different software packages for the 3D rendering of the upper airway anatomy.

## Background

The association between breathing disorders and craniofacial morphology has determined a growing interest in the form and size of the upper airway [1].^2^]. Skeletal openbite, transverse maxillary deficiency, and mandibular growth pattern featuring clockwise rotation, with or without mandibular retrognathia, are often associated with chronical oral breathing [3, 4], leading to a long-face syndrome [5–8]. Airway obstructions can also contribute to the development of obstructive sleep apnea syndrome (OSAS) [9, 10] in both children and adults [11, 12]. This condition is characterized by the appearance of nocturnal symptoms (persistent snoring, sleep breaks, restless sleep and polyuria) and day-time symptoms (drowsiness, headache, asthenia, memory disorders, irritability) which impair patients’ general health condition and quality of life [13–16]. For this reason, a comprehensive and early evaluation of the airway shape and dimensions can be useful in both youngsters and adult subjects [1, 17].

Cone-beam computed tomography (CBCT) has become a widespread method to visualize the upper airway thanks to less radiation dose than traditional computed tomography (CT) [18, 19], and better effectiveness in discriminating the boundaries between soft and hard tissues [20, 21]. In addition, this 3-dimensional (3D) imaging system offers information on cross-sectional areas, volume, and 3D form that cannot be determined by 2-dimensional (2D) images.

The first step to analyze the upper airway in 3-dimension from CT or CBCT is the segmentation process, which means to virtually isolate the structure of interest by removing all the neighboring anatomical regions for better visualization and analysis [22]. Segmentation can be performed manually or by a computer-aided approach. Manual segmentation is performed slice for slice by the operator; then, the software combines the segmented slices to create a 3D volume. However, this procedure is time-consuming and is not convenient for clinical application [17, 23].

The computer-aided approach involves both semi-automatic and fully automatic segmentation of the airway. In the semi-automatic segmentation, the computer detects the boundaries between the air and soft tissues, based on the threshold interval (Hounsfield units) selected by the operator. This procedure is less time-consuming and is not influenced by intra-operator reliability [22]. Instead, fully automated segmentation relies on the application of artificial intelligence (AI), that has shown very encouraging results, and is destined to replace manual and semi-automatic systems in the future. In this regard, the routine usability of AI in clinical settings is still limited due to the sophisticated computer and software equipment required. Thus, so far, the semi-automatic method represents the most efficient tool to obtain virtual reproduction of the upper airway in daily practice settings.

Nevertheless, there is not sufficient evidence in the literature concerning the accuracy of the semi-automatic segmentation of the upper airway. Several software/tools are available for both orthodontists and maxillofacial surgeons, but their performances have not fully investigated yet. The aim of this study was to evaluate the accuracy of five software for the semi-automatic segmentation of the upper airway and to establish if they could be considered alternative to the gold standard (manual segmentation). For this purpose, we referred to a specific 3D digital diagnostic technology that involved volumetric assessment and the surface-to-surface analysis [24–26] of 3D rendered airway models. The null hypothesis was the absence of significant differences in the accuracy of semi-automatic segmentation software compared to manual segmentation.

## Methods

### Study sample

The present study received the approval of the Institutional Ethical Committee of the University of Catania (protocol n. 119/2019/po-Q.A.M.D.I.) and has been carried out following the Helsinki Declaration on medical protocols and ethics.

The study sample consisted of 20 subjects (eleven females, nine males; mean age 27.6 ± 4.6 years old) selected from a larger sample of patients who referred for orthognathic surgery evaluation; therefore, patients included in the study sample had not been subjected to additional radiation for the purpose of the present investigation. The inclusion criteria were as follows: subjects between 18 and 40 years old, good quality CBCT scans, absence of artifacts or distortions, field of view (FOV) including the upper airway to at least the third cervical vertebra. Subjects with craniofacial anomalies, airway pathology, previous orthognathic or craniofacial surgery were excluded.

Patients were scanned with the same CBCT machine (KODAK 9500 3D® Carestream Health, Inc., Marne-la-Vallée, France, 90 kV, 10 mA, 0.2 mm voxel size) and were instructed to maintain the head in natural position, with teeth in maximum intercuspidation, and to refrain from swallowing during the scan period. After scan, the acquired data sets images were saved in Digital Imaging and Communications in Medicine (DICOM) and anonymized to protect patients’ data.

### Step 1: Preliminary definition of Volume of Interest (VOI)

**Fig. 1:**
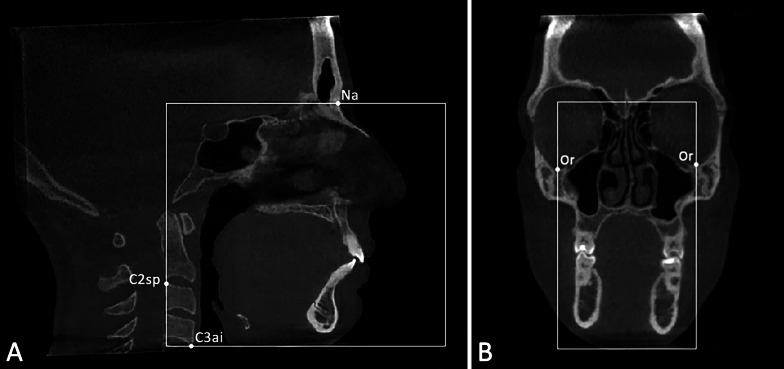
Landmarks and boundaries of the volume of interest (VOI). **A**) Medio-sagittal scan: Na point (most anterior point of the frontonasal junction), C3AI point (most anterior inferior point on the third cervical vertebra) and C2SP point (most superior posterior point on the second cervical vertebra); **B**) Coronal scan: OR points (right and left most inferior point of the orbit)

**Fig. 2:**
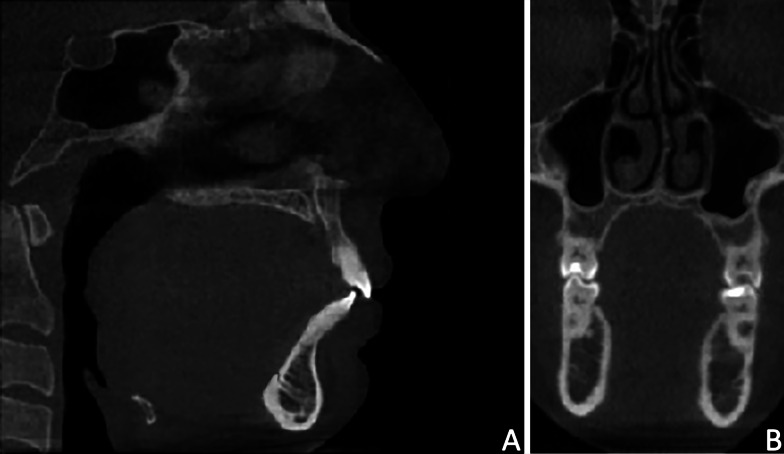
The new cropped DICOM file generated with the exclusion of the slices in over the borders of V.O.I. **A**) Medio-sagittal scan; **B**) Coronal scan

Firstly, the 20 CBCTs were imported into Mimics software (version 21.0; Materialise, Leuven, Belgium) and the Volume of Interest (VOI) was defined by selecting the following reference points: Na point (most anterior point of the frontonasal junction), C3AI point (most anterior inferior point on the third cervical vertebra) and C2SP point (most superior posterior point on the second cervical vertebra) in the medio-sagittal scan (Fig. 1A), and the OR points (right and left most inferior point of the orbit) in the coronal scan (Fig. 1B). The DICOM files with the defined VOI were used to perform both manual and semi-automatic segmentation (Fig. 2A, B). In this regard, the VOI was selected prior to the usage of semi-automatic software, to exclude the error in the definition of the VOI using different software and relative tools.

### Step 2: Upper airway segmentation

**Fig. 3:**
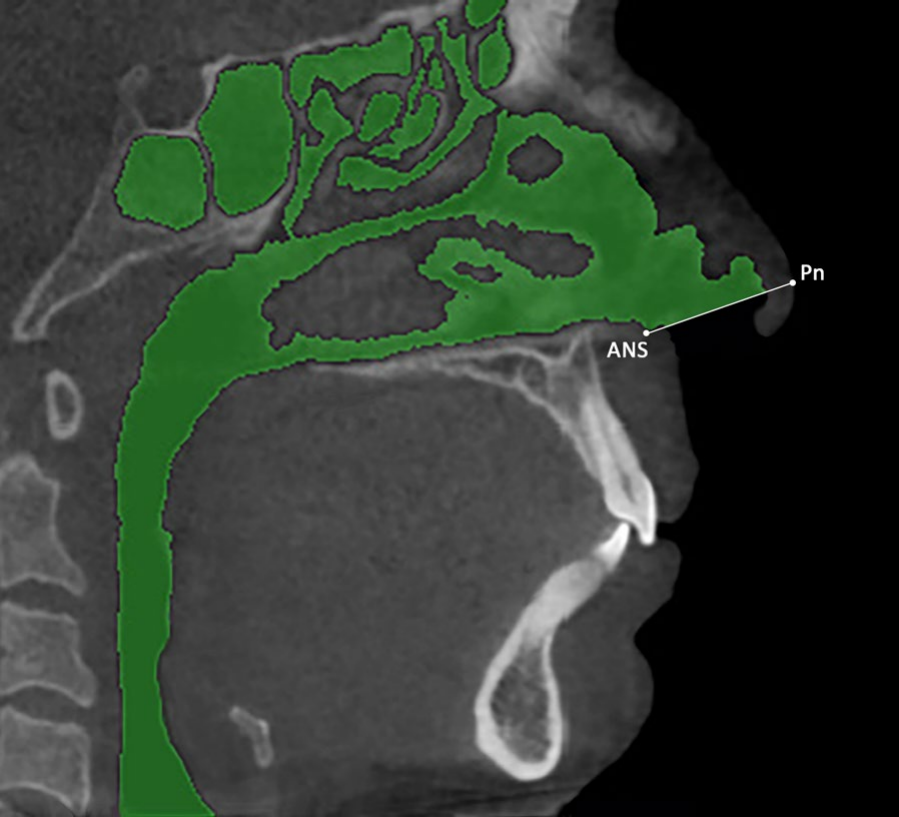
Manual segmentation mask of the upper airway and landmarks of the cutting plane to exclude the lowermost area of the nostrils. ANS = anterior nasal spine; Pn = soft tissue Pronasal point

The Mimics software was used to carry out the manual segmentation of the upper airway, and the 3D models generated were used as gold standard (GS) for the comparative assessments between semi-automatic software. In particular, data for the upper airway boundaries were obtained by a manual slice-by-slice segmentation of the data sets. Afterward, a cutting plane passing for the soft tissue Pronasal point (Pn) and anterior nasal spine (ANS) was generated to allow the exclusion of the lowermost area of the nostrils (Fig. 3).

Five software were used for the semi-automatic segmentation of the upper airway, respectively: Dolphin3D (Dolphin Imaging, version 11.0, Chatsworth, CA, USA), Invesalius (version 3.0.0; Technology center from Informação Renato Archer, Campinas, SP, Brazil), ITK-SNAP (version2.2.0; www.itksnap.org), 3D Slicer (http://www.spler.org) and Seg3D (version 2.2.1, Scientific Computing and Imaging Institute, University of Utah, HTTPS: / /www.sci.utah.edu/cibc-software/seg3d.html). The segmentation of the upper airway was performed using the interactive threshold technique, which means that the operator selected the best threshold interval to display the set of anatomical airway boundaries. The Invesalius and Seg3D software featured a *binary threshold algorithm* [27] while Dolphin 3D, ITK-Snap and 3D Slicer software featured a *region growing algorithm *[27] for the segmentation process. Once the segmentation mask was obtained, the 3D rendered models were generated and exported as an electronic STL ASCII format. Image processing time was calculated for each software tested and data were recorded on a spreadsheet.

### Step 3: Volumetric assessment and model superimposition

**Fig. 4:**
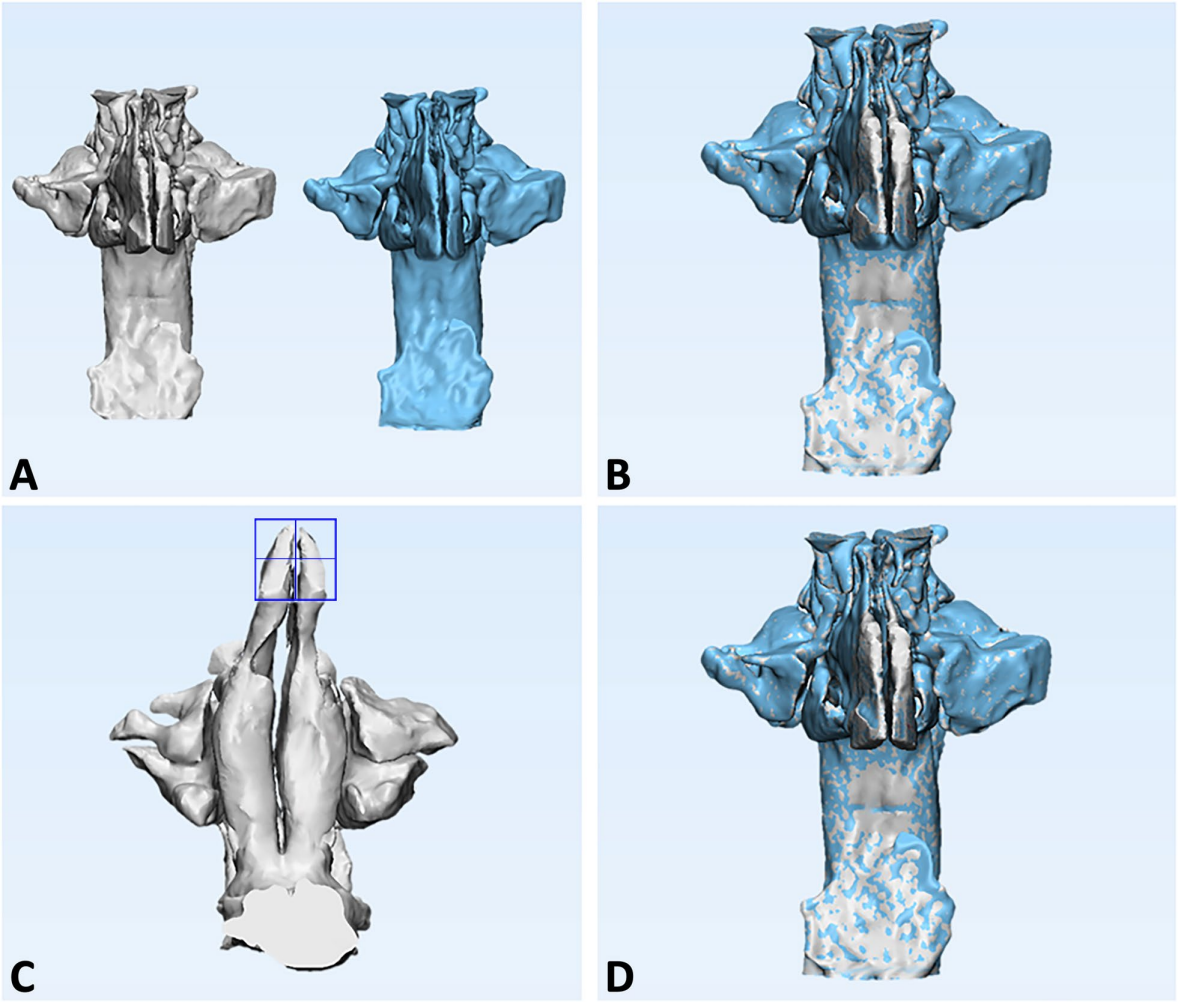
Each 3D upper airway model obtained from semi-automatic segmentation was superimposed to its ground truth model (manual segmentation) in order to reliably remove the lowermost area of the nostrils. **A**, **B**) Superimposition between GS and semi-automatic models, using a global surface-based registration method; **C**, **D**) Cutting plane for exclusion of the lowermost area of the nostrils

The airway 3D models were imported into 3-Matic software (version 13.0; Materialise, Leuven, Belgium) to perform the superimposition between GS and semi-automatic models, using a global surface-based registration (best-fit algorithm) method (Fig. 4A, B). Once the two models were superimposed, a cutting plane was generated by selecting 3 random points on the anterior surface of the GS model. The cutting plane served to exclude the lowermost area of the nostrils that was still represented in the semi-automatic model (Fig. 4C, D). The software also calculated the total volume of the 3D models of the upper airway.

### Step 4: Deviation analysis and surface-to-surface matching technique

**Fig. 5:**
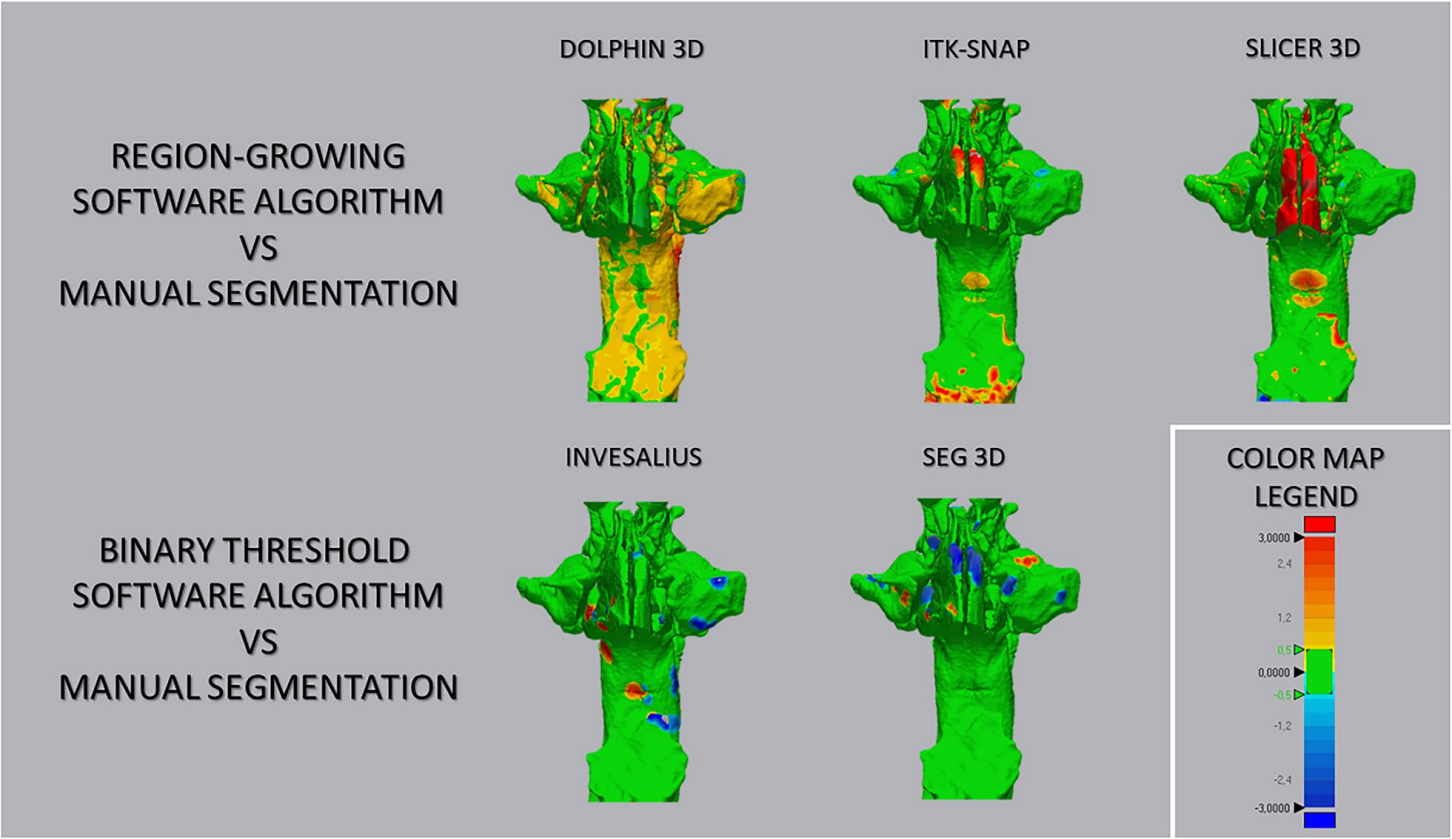
Surface-to-surface matching technique between 3D upper airway models obtained with semi-automatic segmentation and its ground truth model (manual segmentation)

Finally, the surface-based deviation analysis was carried out in the Geomagic Control X software (version 2017.0.0, 3D Systems, Santa Clara, CA, USA) that automatically calculates the mean and maximum values of the linear distances (Euclidean distance) between the surfaces of the two upper airway models. These values were measured across 100% of the surface points. The analysis was complemented by the visualization of the 3D color-coded maps, set at 0.5 mm range of tolerance (green color), to better evaluate and locate the discrepancy between the model surfaces (Fig. 5). Distance values higher than the positive limits (yellow-to-red fields) indicated that the semi-automatic model was wider than the GS, instead distance values smaller than the negative limits indicated that the semi-automatic model was narrower compared to the GS. After the deviation analysis, the percentages of all the distance values within the tolerance range were calculated. These values represented the degree of correspondence between the two models and, therefore, show the surface accuracy of the 3D models of the airway obtained with the tested software (semi-automatic segmentation). All data were recorded on a spreadsheet and used for comparative analyses.

The entire workflow, including segmentation and relative generation of the mask, was carried out by the same experienced operator with 5 years of experience in digital orthodontics (V.R.). The images were re-measured 4 weeks after the last examination, to obtain data for intra-operator reliability assessment, and separate spreadsheets were generated to blind the operator to the previous data. A second expert operator (A.L.G.) also performed the entire workflow in order to obtain data for the assessment of inter-operator reliability.

### Statistical analysis

10 CBCT were randomly selected to preliminary assess sample size power. The analysis revealed that 15 examinations were required to reach the 80% power to detect a mean difference of 5.08 cm^3^ in the volumetric assessment of upper airway between manual segmentation and semi-automatic segmentation (Dolphin Software), with a confidence level of 95% and a beta error level of 20%. According to the inclusion criteria, we were able to include 20 CBCTs, which increased the robustness of the data.

The assessment of normal distribution and equality of variance of the data was performed with Shapiro–Wilk Normality Test and Levene’s test. Since the data were normally distributed and showed homogeneous variance, parametric tests were used to analyze and compare measurements.

The one-way analysis of variance (ANOVA), adjusted with Post-hoc Scheffè test, was used to assess the volumetric differences among the 3D rendered models generated from different software. The same test was used to compare data of the surface matching percentage and of segmentation timing. The Bland–Altman analysis was used to quantify the agreement between semi-automatic models and GS models of the upper airway, and to obtain a precise confidence interval. The same test was also used to assess the intra-observer and inter-observer agreement between first and second measurements. Finally, inter-software reliability (GS vs semi-automatic software) was calculated using the Intraclass Correlation coefficient (ICC), referring to the following score: ICC < 0.50 = poor reliability, ICC = 0.50.-0.75 = moderate reliability, ICC > 0.75 = high reliability [28]. Data were analyzed using SPSS® version 24 Statistics software (IBM Corporation, 1 New Orchard Road, Armonk, New York, USA) with a significance level set at *p* < 0.05.

## Results

According to the one-way analysis of variance (ANOVA), statistically significant differences were found among the volumetric measurements obtained with different software (*p* < 0.001). In this regard, Dolphin 3D was the only semi-automatic software showing statistically significant volumetric differences (*p* < 0.001) compared to the manual segmentation, as assessed by post-hoc analysis tests (Table 1).

**Table 1:** Comparison of the volumetric measurements of upper airways among different software tested

	Sample	Mean (cm^3^)	SD	Confidential Interval		*F*	Significance
				Lower Limit	Upper Limit		
Mimics (a)	20	89.94 (d)	4.76	87.71	92.17	9.125	*p* < 0.001
ITK-Snap (b)	20	92.46 (f)	5.68	89.80	95.12		
Invesalius (c)	20	88.40 (d.e)	4.72	86.19	90.61		
Dolphin 3D (d)	20	96.01 (a,c,f)	6.36	93.03	98.98		
Slicer 3D (e)	20	94.72 (c,f)	5.54	92.12	97.31		
Seg3D (f)	20	86.72 (b,d,e)	5.10	84.34	89.12		

*Significance set at *p* < 0.05 and based on one-way analysis of variance (ANOVA) and Scheffe's post-hoc comparisons tests; a, b, c, d, e, f = identifiers for post-hoc comparisons tests

*SD* standard deviation

**Fig. 6:**
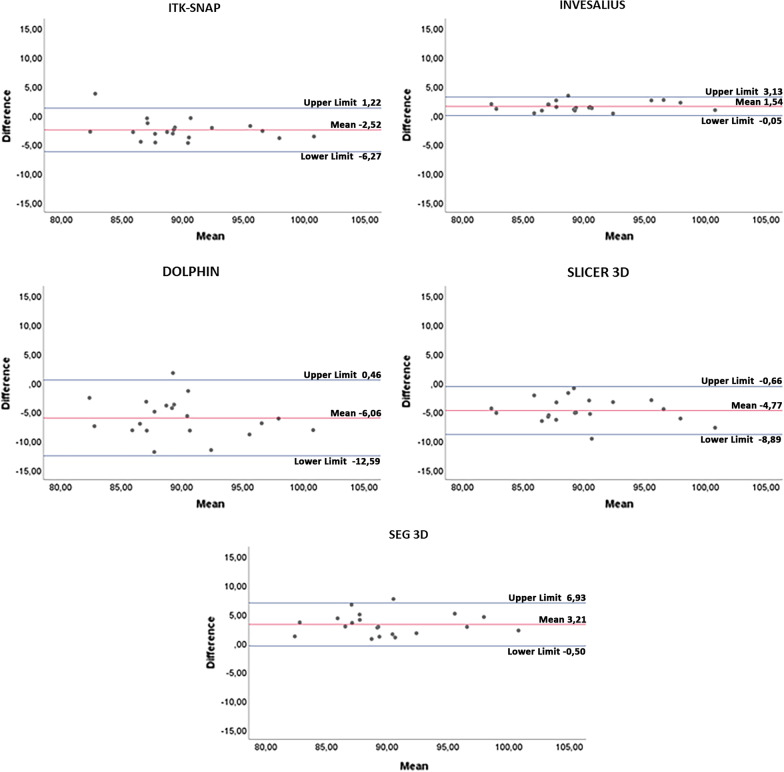
Bland–Altman plot with lines of agreement between manual segmentation and semi-automatic segmentation of the upper airway

The mean bias of volume (cm^3^) and the relative limits of agreement (LOA) were obtained from Bland–Altman analysis for each semi-automatic software (Fig. 6): ITK-SNAP: mean bias =  − 2.52 cm^3^, LOA = 1.22 to – 6.27 cm^3^; Invesalius: mean bias = 1.54 cm^3^, LOA = 3.13 to − 0.05 cm^3^; Dolphin: mean bias =  − 6.06 cm^3^, LOA = 0.46 to − 12.59 cm^3^; Slicer 3D: mean bias =  − 4.77 cm^3^, LOA =  − 0.66 to – 8.89 cm^3^; Seg3D: mean bias = 3.21 cm^3^, LOA = 6.93 to – 0.50 cm^3^. Invesalius and Seg3D showed a statistically significant underestimation of upper airway volume (*p* < 0.001), instead ITK-SNAP, 3D Slicer and Dolphin 3D showed a statistically significant overestimation of the same data (*p* < 0.001). Almost all points were evenly distributed above and below the mean difference, with limited scattering and within the calculated range of agreement [29]. Although differences between semi-automatic software and GS were observed, volumetric data showed excellent reliability, with coefficient values ranging from 0.904 to 0.993 (Table 2).

**Table 2:** Inter-software reliability (Gold Standard vs semi-automatic software) of upper airway segmentation

	ITK-Snap	Invesalius	Dolphin 3D	Slicer 3D	Slicer 3D
*Mean*	92.61	89.28	95.99	95.51	86.95
*SD*	5.27	5.64	6.03	6.63	5.31
ICC	0.966	0.993	0.904	0.957	0.962

*ICC* Intraclass correlation coefficient

**Fig. 7:**
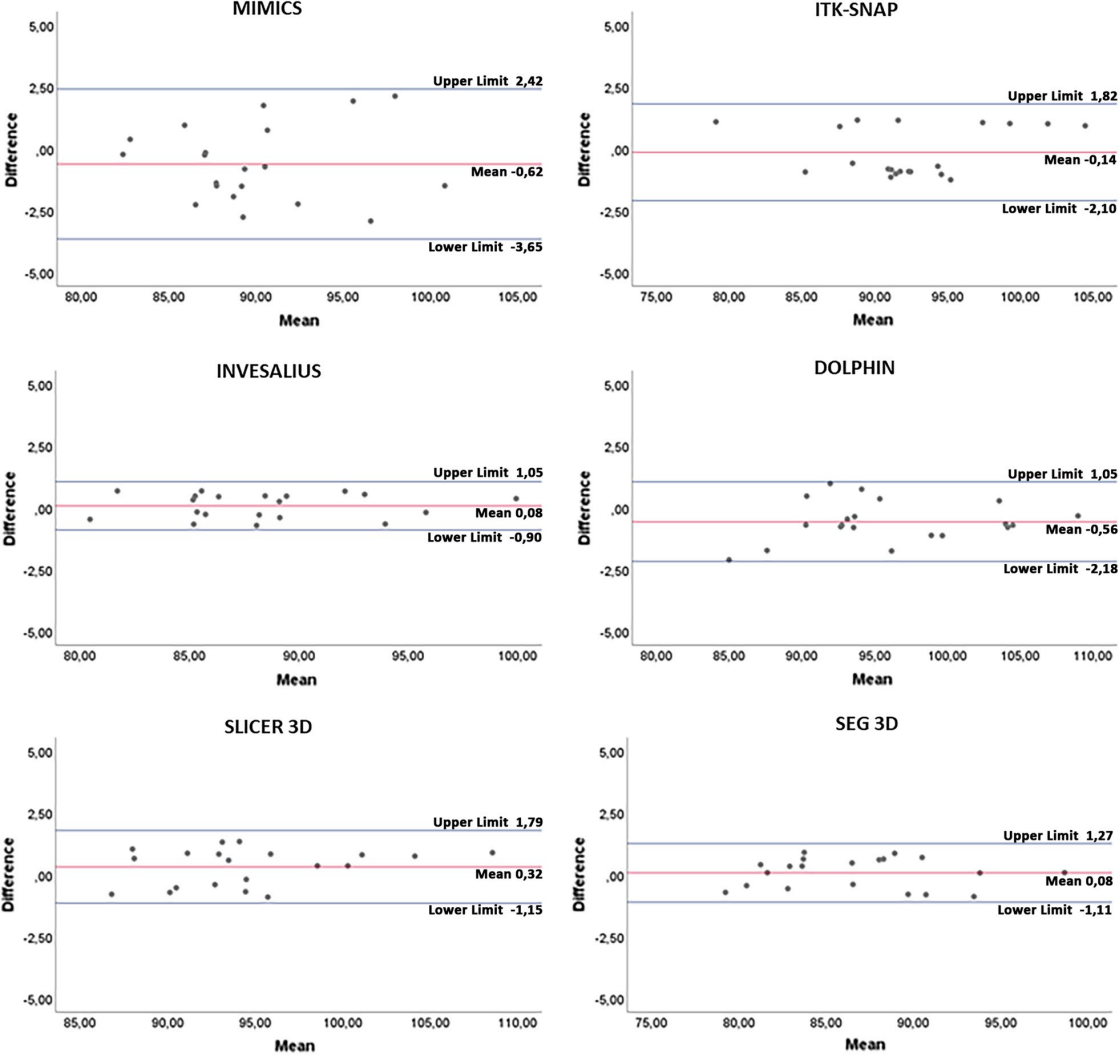
Bland–Altman plot with lines of agreement between first and second intra-operator readings of semi-automatic segmentation of the upper airway

**Fig. 8:**
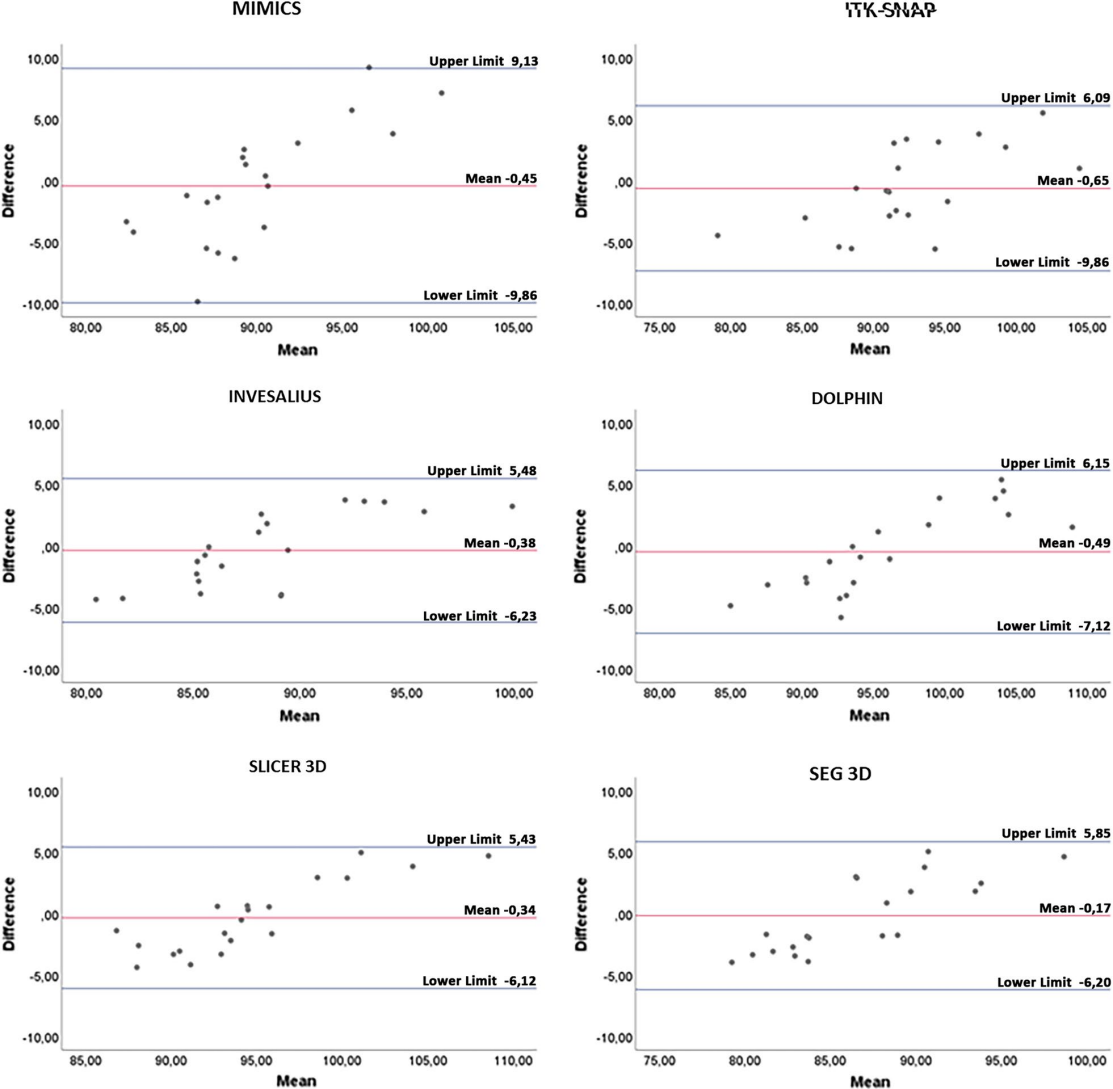
Bland–Altman plot with lines of agreement between first and second inter-operator readings of semi-automatic segmentation of the upper airway

Concerning intra-operator agreement, the mean bias of volume (cm^3^) and the relative limits of agreement (LOA) obtained from Bland–Altman analysis were (Fig. 7): Mimics: mean bias =  − 0.62 cm^3^, LOA = 2.42 to – 3.65 cm^3^; ITK-SNAP: mean bias =  − 0.14 cm^3^, LOA = 1.82 cm^3^ to – 2.10 cm^3^; Invesalius: mean bias = 0.08 cm^3^, LOA = 1.05 to – 0.90 cm^3^; Dolphin: mean bias =  − 0.56 cm^3^, LOA = 1.05 to – 2.18 cm^3^; Slicer 3D: mean bias = 0.32 cm^3^, LOA = 1.79 to – 1.15 cm^3^; Seg3D: mean bias 0.08 cm^3^, LOA = 1.27 to – 1.11 cm^3^. Instead, for inter-operator agreement, the mean bias of volume (cm^3^) and the relative limits of agreement (LOA) obtained from Bland–Altman analysis were (Fig. 8): Mimics: mean bias =  − 0.45 cm^3^, LOA = 9.13 to – 9.86 cm^3^; ITK-SNAP: mean bias – 0.65 cm^3^, LOA = 6.09 to – 9.86 cm^3^; Invesalius: mean bias – 0.38 cm^3^, LOA = 5.84 to – 6.23 cm^3^; Dolphin: mean bias – 0.49 cm^3^, LOA = 6.15 to – 7.12 cm^3^; Slicer 3D: mean bias – 0.34 cm^3^, LOA = 5.43 to – 6.12 cm^3^; Seg3D: mean bias 0.17 cm^3^, LOA = 5.85 to – 6.20 cm^3^. The mean difference between the two readings was close to 0, and not statistically significant (*p* > 0.05), for all software tested. These data would suggest that no systematic bias would affect inter-operator intra-operator reliability in the present investigation. Almost all points were evenly distributed above and below the mean difference, with limited scattering and within the calculated range of agreement [29].

Statistically significant differences (*p* < 0.001) were found among the matching percentages recorded between the GS model and each model obtained from semi-automatic software, according to the one-way analysis of variance (ANOVA). In particular, Dolphin 3D and Invesalius showed, respectively, the lower (78.25%) and the higher (90.05%) matching values with the manual 3D rendered model (Table 3).

**Table 3:** Comparison of the matching percentages obtained superimposing the semi-automatic model of the upper airways with the ground truth (manual segmentation), according to the deviation analysis

	Sample	Mean (%)	SD	Confidential interval		*F*	Significance
				Lower limit	Upper limit		
ITK-Snap (a)	20	84.44 (b,c)	4.84	82.18	86.71	25.117	*p* < 0.001
Invesalius (b)	20	90.05 (a,c,d)	3.14	88.57	91.52		
Dolphin 3D (c)	20	78.26 (a,b,d,e)	5.40	75.73	80.78		
Slicer 3D (d)	20	82.08 (b,c,e)	3.24	80.56	83.60		
Seg3D (e)	20	87.36 (c,d)	3.21	85.85	88.86		

*Significance set at *p* < 0.05 and based on one-way analysis of variance (ANOVA) and Scheffe's post-hoc comparisons tests; a, b, c, d, e = identifiers for post-hoc comparisons tests

*SD* Standard Deviation

Concerning image processing time, statistically significant differences were found only between Invesalius and ITK-SNAP (*p* < 0.05), with an average segmentation timing, respectively, of 12.11 min and 18.05 min, according to the one-way analysis of variance (ANOVA) and post-hoc comparison tests (Table 4).

**Table 4:** Comparison of the image processing time for upper airway segmentation

	Sample	Mean time (min)	SD	Confidential interval (min)		*F*	Significance
				Lower Limit	Upper Limit		
ITK-Snap (a)	20	18.05 (b)	6.54	14.99	21.11	3.280	*p* < 0.05
Invesalius (b)	20	12.11 (a)	3.29	10.57	13.65		
Dolphin 3D (c)	20	16.43	5.48	13.87	19.00		
Slicer 3D (d)	20	14.81	7.09	11.49	18.13		
Seg3D (e)	20	14.79	3.86	12.99	16.61		

*Significance set at *p* < 0.05 and based on one-way analysis of variance (ANOVA) and Scheffe's post-hoc comparisons tests; a, b, c, d, e, f = identifiers for post-hoc comparisons tests

*SD* standard deviation

## Discussion

The reliability of the three-dimensional analysis of upper airways relies on the accuracy of the segmentation process. There are several software available for the 3D elaboration of the images obtained from CBCT, most of them including semi-automatic segmentation tools [27]. Recently, artificial Intelligence (AI) systems have been validated for the segmentation of the airway with the aim to maximize the efficiency and reducing the variability related to the operator [17]; however, fully automatic segmentation technologies based on AI require very elaborate software and are still restricted to research environments. Thus, from a clinical perspective, it is important to evaluate the accuracy of semi-automatic software usable to clinicians, if we consider that the 3D assessment of the upper airway provides useful data for the diagnosis of breathing or sleeping disorders [30]. In this study, we tested the accuracy of the upper airway segmentation performed with semi-automatic software that are widespread among orthodontists and maxilla-facial surgeons (Dolphin 3D) or that are open-source or commonly used in medicine or biomedical engineering (ITK-SNAP, Invesalius, 3D Slicer, Seg3D, Mimics).

To perform this analysis, we used the 3D airway models generated from manual segmentation as the ground truth of the investigation. In fact, in the absence of the physical anatomical structure or its realistic reproduction obtained from laser scanning, the manual segmentation represents the ground truth anatomical reference and the gold standard for 3D rendering, since it allows the detection of areas with no-well defined boundaries due to low contrast and proximity to other structures [17, 26, 31, 32]. The image scans involved in the present study were obtained from the same CBCT, using the same acquisition parameters, patient positioning and management, volume reconstruction, and DICOM export [33]. This allowed for a rigorous control of the factors affecting the accuracy of the 3D model rendering prior to the segmentation process [34].

According to the present results, the volumetric rendering of the upper airway obtained with Invesalius showed the lower values of accuracy error, with a volumetric bias from the reference manual segmentation (Mimics) of 1.54 cm^3^, followed by ITK-SNAP (2.52 cm^3^), Seg3D (3.21 cm^3^), 3D Slicer (4.77 cm^3^) and Dolphin 3D (6.07 cm^3^). On average, Dolphin 3D, ITK-SNAP and 3D Slicer overestimated the volume generated with manual segmentation (GS), instead Invesalius and Seg3D underestimated the volume of the upper airway (Table 1). Figure 6 shows the limits of agreement recorded with each semi-automatic software tested in the present study.

Although accuracy data are critical, it remains questionable if the mean differences (bias) and the limits of agreement (LOA) recorded for each software are relevant from the clinical and diagnostic perspective, considering that there is no norm for airway volumes [35]. In this regard, the airway volume is extremely variable, depending on head posture, breathing stage and anatomical complexity, which makes difficult to establish a volumetric cut-off for normal condition [36]. At the same time, the semi-automatic software showed excellent reliability compared with manual segmentation (Table 2). This means that, despite volumetric data were different, they were proportionally equivalent. As consequence and according to our findings, semi-automatic software could replace manual segmentation especially in the absence of normal values for the upper airway [35].

Volumetric data do not provide a qualitative assessment of the accuracy of the rendered models, since they do not allow the discernment between matching and un-matching area of two models generated from the same ground truth anatomy. Therefore, to deeply investigate the accuracy of the semi-automatic segmentation, we performed the superimposition between semi-automatic and manual 3D models. Afterward, the surface-to-surface matching technique was used to detect the differences in shape between the two airway models (semi-automatic vs manual segmentation), according to a consolidated methodology [1, 37, 38]. Most of the semi-automatic software showed good surface correspondence with the manual segmentation, ranging from 82.08% (3D Slicer) to 90.05% (Invesalius), instead Dolphin software showed the lowest surface agreement (78.26%) (Table 3).

The color-coded map showed that the dis-matched area between manual segmentation and semi-automatic segmentation was located at the most anterior nasal region of the airway, specifically at the boundaries between the nasal mucosa and the airway (Fig. 5). This finding could be explained considering the intrinsic complexity in the reconstruction of this anatomical region from CBCT scans, due to the low-contrast representation of the involved tissues (mucosa and airway), that may have generated biases and caused overestimation or underestimation of the airway [39]. Nevertheless, the models generated with Dolphin showed an extent area of mismatching that would result in a wider surface compared with the manual segmentation (in the yellow–red fields).

Being all other variables equal [31, 40], the factor that could have significantly influenced the generation of the masks in this study is the performance of the threshold selection algorithm. Semi-automatic segmentation of the airway was performed using the interactive threshold technique, which means that the operator selected the best threshold interval to better visualize the anatomical boundaries of the upper airway. This process depends on the software algorithm, the spatial resolution and contrast of scanning, the thickness of mucous membranes and bone structures and, above all, on the ability and technical experience of the operator [34]. In this regard, the tested software present different semi-automatic segmentation algorithms. Dolphin 3D, ITK-SNAP and 3D Slicer software run the *region growing algorithm*, in which the user selects the seed points for 3D rendering, based on the threshold set, after selecting the region of interest (ROI). Instead, Invesalius and Seg3d software feature a *threshold-based algorithm*, which relies on the visual discrimination of the structures and the definition of threshold level. The differences in the active role of the operator with both systems may be contributed to the different trends found in this study, i.e., the overestimation (3D Dolphin software, ITK-SNAP, 3D Slicer) and underestimation (Invesalius and Seg3d) of the 3D rendered airway volumes (Table 1, Fig. 6), particularly considering the proximity of two different structures (mucosa and air) with an intense similar radio-opacity [31, 41].

In the present study, the threshold level was different among the semi-automatic software. It could be argued that interactive threshold technique is influenced by human skills, and consequently, it is less reliable compared to fixed threshold [27] which eliminates operator subjectivity in boundary selection. However, to reach a comprehensive evaluation of software performance, it must be bear in mind the intrinsic differences between TC and CBCT in assessing density unit when considering these two threshold selection systems. In CT scans, Hounsfield Unit (HU) is proportional to the degree of x-ray attenuation and it is allocated to each pixel to show the image that represents the density of the tissue. In CBCTs, the degree of x-ray attenuation is shown by gray scale (voxel value) which are presented as HUs; however, these measurements are not true HUs [42]; instead, they are adapted to the gray scale in a post-processing stage. Also, they can be different among different CBCTs equipment. Thus, the HU fixed threshold is ideal when assessing software performance based on TC images. When evaluating CBCT scans, the fixed threshold could bias the comparative evaluation of different software algorithms in identifying, matching, and filling specific areas [27], especially those with complex morphology and/or low-contrast resolution.

Another strength of our investigation is that we included the nasopharynx in the 3D rendering process, while previous studies [27, 36] evaluated only the three-dimensional reconstruction of the oropharynx region to facilitate the comparative assessment of software performance. Considering the complexity of the nasopharynx, which is characterized by thin and curvy bone laminae (septum, ethmoidal cells, turbinates and medial wall of maxillary sinus) and devious soft-tissue materials, the present study provides new and deeper evidence on the potential of semi-automatic software in segmenting the upper airway. Moreover, the preliminary definition of VOI (Step 1), the anterior cutting plane generated on Mimics software (Step 2) and its reproduction on 3-Matic software (Step 3) allowed a consistent definition of upper airways model boundaries and to perform superimposition and surface-to-surface analysis. By this method, it was possible to integrate volumetric data with surface analysis, identifying those area that mismatched with the ground truth model. Further comparative studies, involving similar technologies, are warmly encouraged to ensure the validity of the diagnosis of the upper airway.

Time-to-segment (efficiency) is another parameter that should be taken into account for in-office applications of anatomic 3D rendering. According to our findings, Invesalius showed less time to segment the upper airway compared to the other tested software (Table 4). With semi-automatic software, the key steps that mainly influence segmentation timing are the manual touch-up and mesh creation [43]. In the present study, the manual touch-up procedure, necessary to fix the boundaries of the upper airway and/or stray pixels during the refinement of the segmentation mask, was more time-consuming with 3D Slicer and Seg 3D; also, Dolphin 3D and ITK-SNAP did not allow to modify the generated mesh and any stray pixel must be fixed by reorganizing the selected seeding points.

Considering the comparative assessment of the software accuracy and the efficiency of the present study, Invesalius would represent the best alternative for manual segmentation of the upper airway. However, a slight longer learning curve is required when compared to Dolphin 3D, which is designed for orthodontists and maxilla-facial surgeons and features a simple and user-friendly interface. Table 5 shows a detailed overview of the characteristics of the semi-automatic software tested in the present study.

**Table 5:** Advantages and disadvantages of the 5 imaging softwares used in the present study

Name	Vantages	Disadvantages
*ITK-Snap*	Free source	Not user-friendly interface
	Good threshold sensitivity	Designed for usage in medicine
	Tools for checking and correction of segmentation mask in 2D views	
	Compatibility with multiple operating systems (Windows, Mac OS X, Linux)	
*Slicer 3D*	Free source	Not user-friendly interface
	Good threshold sensitivity	Lack of 2D correction tools for segmentation mask
	Compatibility with multiple operating systems (Windows, Mac OS X, Linux)	
*Dolphin 3D*	User-friendly interface	Not free source
	Designed for orthodontists and maxillofacial surgeons	In regions with complex morphology (ethmoid cells) the segmentation algorithm does not distinguish thin osseous laminae from air
	Good threshold sensitivity	Compatibility with single operating system (Windows)
*Invesalius*	Free source	Not user-friendly interface
	Good threshold sensitivity	
	Compatibility with multiple operating systems (Windows, Mac OS X, Linux)	
*Seg3D*	Free source	Not user-friendly interface
	Tools for checking and correction of segmentation mask in 2D views	Deficient threshold sensitivity
		Compatibility with single operating system (Windows)

### Limitations

- The present findings should not be generalized since the same CBCT apparatus has been used for all acquisitions. In this regard, future studies should evaluate software performance even in relation to images obtained from different CBCTs.
- Since the study sample consisted of subjects with a skeletal maxillary transverse deficiency, referring to surgically assisted expansion therapy, it is not representative of a normal population. However, this should not be considered a major weakness considering that the study was limited to the comparative evaluation of software performance.
- Finally, the small sample size could represent a limitation of the present study considering the significant anatomical variations of the upper airways among general population.

## Conclusions

- Among the software tested, Invesalius would represent the best alternative to the manual segmentation of the upper airway in terms of accuracy and efficiency performances.
- Different semi-automatic segmentation algorithms could generate different patterns of inaccuracy error (underestimation/overestimation) of the upper airway models. Thus, it is unreasonable to expect volumetric agreement among different software packages for the 3D rendering of the upper airway anatomy.
- The dis-matched area between manual segmentation and semi-automatic segmentation was located at the most anterior nasal region of the airway, specifically at the boundaries between the nasal mucosa and the airway.

## Data Availability

The datasets used and analyzed during the current study are available from the corresponding author on reasonable request.
